# Antigen 85B peptidomic analysis allows species-specific mycobacterial identification

**DOI:** 10.1186/s12014-017-9177-6

**Published:** 2018-01-08

**Authors:** Wei Zhang, Qingbo Shu, Zhen Zhao, Jia Fan, Christopher J. Lyon, Adrian M. Zelazny, Ye Hu

**Affiliations:** 1 Department of Respiratory Medicine, Shengjing Hospital of China Medical University, 36 Sanhao Street, Shenyang, 110004 Liaoning Province China; 2 Virginia G. Piper Biodesign Center for Personalized Diagnostics, Arizona State University Biodesign Institute, Tempe, AZ 85287 USA; 3 School of Biological and Health Systems Engineering, Arizona State University, Tempe, AZ 85287 USA; 4 Department of Laboratory Medicine, Clinical Center, National Institutes of Health, Bethesda, MD 20892 USA

**Keywords:** Tuberculosis, Nontuberculous mycobacteria, Antigen 85B, Diagnosis, Liquid chromatography–tandem mass spectrometry

## Abstract

**Background:**

Nontuberculous mycobacteria (NTM)-mediated infections are a growing cause of worldwide morbidity, but lack of rapid diagnostics for specific NTM species can delay the initiation of appropriate treatment regimens. We thus examined whether mass spectrometry analysis of an abundantly secreted mycobacterial antigen could identify specific NTM species.

**Methods:**

We analyzed predicted tryptic peptides of the major mycobacterial antigen Ag85B for their capacity to distinguish *Mycobacterium tuberculosis* and three NTM species responsible for the majority of pulmonary infections caused by slow-growing mycobacterial species. Next, we analyzed trypsin-digested culture supernatants of these four mycobacterial species by liquid chromatography–tandem mass spectrometry (LC–MS/MS) to detect candidate species-specific Ag85B peptides, the identity of which were validated by LC–MS/MS performed in parallel reaction monitoring mode.

**Results:**

Theoretical tryptic digests of the Ag85B proteins of four common mycobacterial species produced peptides with distinct sequences, including two peptides that could each identify the species origin of each Ag85B protein. LC–MS/MS analysis of trypsinized culture supernatants of these four species detected one of these species-specific signature peptides in each sample. Subsequent LC–MS/MS analyses confirmed these results by targeting these species-specific Ag85B peptides.

**Conclusions:**

LC–MS/MS analysis of Ag85B peptides from trypsin-digested mycobacterial culture supernatants can rapidly detect and identify common mycobacteria responsible for most pulmonary infections caused by slow-growing mycobacteria, and has the potential to rapidly diagnose pulmonary infections caused by these mycobacteria through direct analysis of clinical specimens.

**Electronic supplementary material:**

The online version of this article (10.1186/s12014-017-9177-6) contains supplementary material, which is available to authorized users.

## Background

*Mycobacterium tuberculosis* (*Mtb*) infections remain a major worldwide health threat, but the prevalence of debilitating pulmonary disease caused by nontuberculous mycobacteria (NTM), which are ubiquitous environmental pathogens, has been on the rise for decades [1–3] and is an important cause of morbidity in North America [4]. Population-based data from North America, Europe and Australia, and large tertiary care facility-based studies in East Asia reveal that the incidence of NTM-related pulmonary disease continues to increase [1], with estimates ranging from 15.5 to 26.7 cases per 100,000 adults > 50 years of age [4]. In South African and Asian countries, NTM infections also comprise a significant fraction of suspected TB and multidrug-resistant TB (MDR-TB) cases [1], with NTM being responsible for 4.2–30% of suspected TB cases [5–7] and 18–27% of suspected “chronic” MDR-TB cases [8–11].

Accurate identification of the mycobacteria responsible for a pulmonary infection at the species/complex level is critical for the selection of the appropriate antimicrobial therapy. This remains challenging, however, since *Mtb* and NTM patients may present with similar symptoms and nonspecific radiographic findings [12].

*Mtb*-specific diagnostic assays, such as IFNγ release assays (IGRAs), can detect *Mtb* exposure, but cannot distinguish between latent and active disease, and have received negative policy recommendations for diagnosis of active *Mtb* [13, 14]. Systematic review has also found that most IGRA results remain positive at the end of treatment [15]. Additionally, IGRAs can cross-react with antigens of *M. kansasii* and other NTM species [16, 17], increasing the odds of a misdiagnosis. Molecular assays for *Mtb* diagnosis, such as the Xpert MTB/RIF assay endorsed by the World Health Organization, allow PCR-based detection of *Mtb* DNA in clinical specimens for rapid, sensitive and specific diagnosis [13], but cannot distinguish between viable and non-viable *Mtb* bacilli.

Acid-fast bacilli (AFB) smears commonly used as a first, rapid assay for diagnosis of mycobacterial infection are relatively insensitive and cannot distinguish between *Mtb* and NTM. Culture remains the gold standard for laboratory diagnosis of mycobacterial infections but requires up to 6 weeks for completion as well as significant technical expertise and analysis time to produce accurate microorganism identifications. Routine use of liquid media for mycobacterial cultures allows early detection of mycobacteria in clinical specimens, but identification of the mycobacterium species present in positive broth cultures requires the use of costly commercial probes or time-consuming molecular methods. Matrix-assisted laser desorption ionization-time of flight mass spectrometry (MALDI-TOF MS) allows rapid and accurate species-specific identification of mycobacterial colonies from solid media and is becoming increasingly popular; however, its performance with positive mycobacterial broth cultures is dramatically hindered by low microbial biomass, the presence of more than one microorganism and/or proteinaceous material.

In contrast to *Mtb*, there are no commercially available, FDA-approved molecular assays for diagnosis of NTM infections from clinical samples, and multiple assays would be required to cover the spectrum of clinically relevant NTM. Some large commercial or academic centers have laboratory-developed methods for one (i.e. M. *avium*) or multiple NTM species; however, these assays differ among these centers and are not standardized, limiting their broader use by the clinical microbiology community. There are thus no satisfactory methods available for rapid and accurate NTM diagnosis.

LC–MS/MS represents an attractive means of identifying mycobacterial species using clinical or culture samples, since it can discriminate peptides derived from highly homologous proteins, such as virulence factors actively secreted by both *Mtb* and NTM. Antigen 85B (Ag85B) is an attractive candidate for this approach, since it represents the major secreted protein in the Ag85 complex, which plays a key role in mycobacterial physiology [18, 19]. Ag85 proteins can also be detected in sera [20–22], sputa [23] and cerebrospinal fluid (CSF) [22] and have been proposed as markers for *Mtb* diagnosis [24]. However, antibodies to *Mtb* Ag85 proteins used in diagnostic assays can cross-react with Ag85 homologues expressed by other mycobacterial species [25]. We now present evidence that LC–MS/MS analysis of mycobacterial Ag85B tryptic digests can identify prominent mycobacterial species involved in pulmonary infections.

## Methods

### Sequence homology analysis of Ag85B proteins from common mycobacterial species

**Fig. 1:**
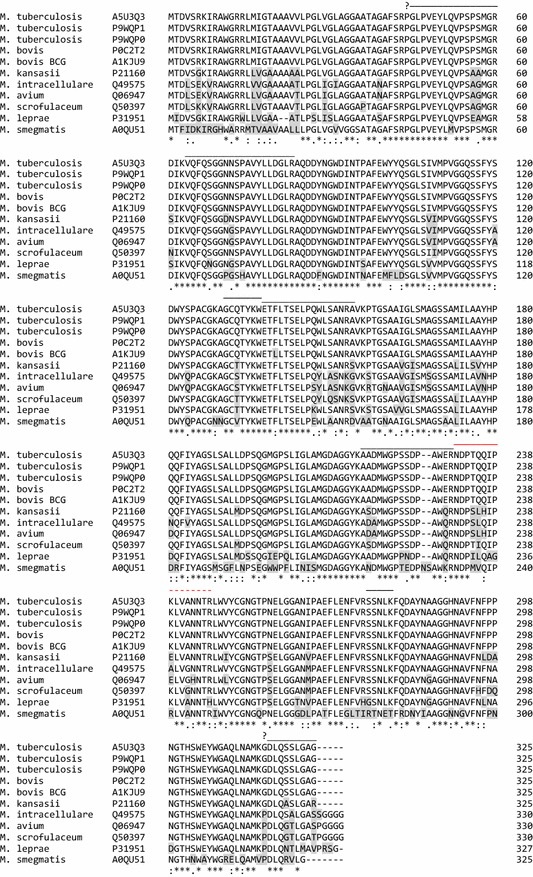
Sequence alignment of reviewed UniProtKB Ag85B protein sequences entries. Shaded amino acids indicate divergence from the consensus sequence of the Mycobacterium tuberculosis complex or the consensus sequence of all proteins with aligned sequence at that position. Sequences are segregated vertically into three groups: members of the mycobacterium tuberculosis complex (5), common pulmonary NTM (3), and other NTM causing human disease (3). Symbols indicate positions with complete conservation (*) or conservation between groups of amino acids with strongly (:) or weakly (.) similar properties. Lines above the sequence indicate observed and/or predicted tryptic peptides, with red font denoting the NDPTIQQK peptide and its variant extension (dashed symbols) in other species, and amino terminal quesiton marks (?) denoting peptide sequences where one or more species contains an amino terminal proline reside expected to suppress cleavage

Querying the UniProt protein sequence database using the criteria “Ag85B”, “mycobacterium”, and selecting only those sequences with reviewed status and annotations scores ≥ 3 identified 11 full-length mycobacterial Ag85B protein sequences. These included entries from three *Mtb* strains (ATCC 25177/H37Ra and 25618/H37Rv and CDC 1551/Oshkosh), two *M. bovis* strains (ATCC BAA-935 and BCG/Pasteur 1173P2), and single entries from *M. avium*, *M. kansasii*, *M. scrofulaceum*, *M. intracellulare* (ATCC 13950), *M. leprae TN,* and *M. smegmatis* (ATCC 700084/mc (2)155). No strain information was available for the *M. avium*, *M. kansasii*, and *M. scrofulaceum* Ag85B protein entries. Clustal Omega (http://www.uniprot.org/align/) was used to individually align the Ag85B sequence of *Mtb* ATCC 25177/H37Ra against each of the other 10 Ag85B sequence entries to determine percent identity and the number of identical and similar amino acids at aligned sequence positions (Fig. 1).

### In silico analysis of Ag85B tryptic peptides

FASTA files of the UniProt *Mtb* ATCC 25177/H37Ra, *M. avium*, *M. intracellulare* (ATCC 13950) and *M. kansasii* Ag85B peptide sequences entries were imported into the Skyline software package and analyzed for tryptic cleavage sites, using default criteria for cleavage site assignment, with the exception of allowing predicted cleavage sites to occur before proline residues.

### Mycobacterial growth conditions and culture supernatant collection

*Mycobacterium tuberculosis* H37Ra, *M. avium* (ATCC 35717), *M. intracellulare* (ATCC 13950) and *M. kansasii* (ATCC 12478) were obtained from the American Type Culture Collection (ATCC, Manassas, VA). A 10 µL loop was used to inoculate mycobacterial colonies into 10 ml of protein-free Sauton’s broth (Himedia, West Chester, PA). Cultures were grown at 37 °C in a shaker incubator (100 rpm) for 22 days, then centrifuged at 16,000×*g* for 3 min, after which supernatants were removed, sterilized with a 0.22 μm filter (Millipore, Billerica MA) and stored at − 80 °C until use. Supernatant protein concentrations were determined with a BCA protein assay reagent kit (Pierce) using bovine serum albumin as the standard.

### Tryptic digestion of mycobacterial culture supernatants

Mycobacterial culture media supernatant aliquots (100 μL) were incubated for 40 min at room temperature with 5 mM Tris (2-carboxyethyl) phosphine to reduce disulfide bonds, then incubated for 40 min at RT with 55 mM iodoacetamide to alkylate cysteine residues, after which samples were protected from light. Samples were then digested using an optimized variant of a published microwave-assisted tryptic protein digestion protocol [26, 27]. Briefly, after addition of trypsin (Promega) to reaction mixtures at a 1:20 trypsin-to-protein mass ratio, samples floating in cold water were irradiated at 240 W for 5 min in a 1200 W microwave oven, with three repetitions, after which samples were incubated at 37 °C for 14 h. Samples were processed with SCX Ziptips (Millipore) to remove detergent and salt, concentrated by vacuum centrifugation, dissolved in 10 μL of 0.1% FA and then centrifuged at 16,000×*g* for 20 min, after which 7 μL samples were injected for LC–MS/MS analysis.

### LC–MS/MS for peptide sequencing and analysis of MS data

Peptide analysis was performed on a nano-LC UltiMate 3000 HLPC column coupled with a LTQ Velos Pro mass spectrometry system (Thermo Fisher Scientific, Waltham, MA, USA). Peptides were separated using EASY-Spray™ C18 LC Columns (15 cm × 75 μm I.D., 3 μm particle size) with a 0.3 μL/min flow rate and 120 min and 30 min linear gradients of 0.1% FA/5–30% acetonitrile for the data dependent acquisition (DDA) and parallel reaction monitoring (PRM) modes, respectively. For DDA, full MS scans were acquired over the *m/z* 200–2000 range, and the 10 most intense peaks were fragmented with a collision energy of 30 and their product ions analyzed to determine the sequence of the precursor ion. Spectra were searched against the actinobacteria (class) protein database in SwissProt using Mascot (version 2.3, Matrix Science, London, UK) using the following criteria: trypsin digestion with a maximum of 2 missed cleavage sites, precursor and product mass tolerances of 2 and 0.5 Da, cysteine carbamidomethylation as a fixed modification and methionine oxidation as a dynamic modification. After peptide identification, [M + H]^2+^ precursor ions corresponding to *Mtb* NDPTQQIPK (*m/z* 520.77), *M. avium* NDPSLHIPELVGHNTR (*m/z* 899.96), *M. intracellulare* NDPSLQIPALVGNNTR (*m/z* 854.95) and *M. kansasii* NDPSLHIPELVANNTR (*m/z* 895.46) precursor and product ions (Additional file 1: Table S1) were applied as targets for MS analysis in PRM mode. Raw data were analyzed against the SwissProt actinobacteria (class) protein database using Mascot software embedded in Proteome Discoverer software (version 1.3, Thermo Scientific) to perform a match between the theoretical and experimental spectra, score these results and determine the false discovery rate using a target decoy control. Parameters used to generate the theoretical spectra were trypsin digestion with no missed cleavage sites, precursor and product mass tolerances of 2 and 0.5 Da.

## Results

### Ag85B is highly conserved among mycobacterial species

A search of the UniProt protein sequence database using the search term Ag85B identified 11 reviewed entries corresponding to Ag85B sequence from three strains of *Mtb,* two strains of *M. bovis, and* six NTM species (*M. avium*, *intracellulare*, *kansasii*, *smegmatis*, *scrofulaceum*, and *leprae*). Ag85B proteins from the three *Mtb* strains and *M. bovis* ATCC BAA-935—all of which cause tuberculosis—demonstrated perfect sequence identity, while Ag85B from the non-virulent *M. bovis* BCG/Pasteur 1173P2 strain revealed a single amino acid mismatch. Ag85B sequences from the six NTM species also revealed significant full-length homology to *Mtb* Ag85B, most demonstrating 89.5–81.5% sequence identity, with only *M. smegmatis* Ag85B revealing less than 80% homology (Table 1), although conserved Ag85 sequence regions demonstrated uneven distribution in this alignment (Fig. 1).

**Table 1:** Sequence homology among reviewed Ag85B UniProt protein entries

Mycobacterium species	Entry	Identity (%)
*M. tuberculosis* ATCC 25177/H37Ra	A5U3Q3	100.00
*M. tuberculosis* ATCC 25618/H37Rv	P9WQP1	100.00
*M. tuberculosis* CDC 1551/Oshkosh	P9WQP0	100.00
*M. bovis ATCC BAA*-*935*	P0C2T2	100.00
*M. bovis* BCG/Pasteur 1173P2	A1KJU9	99.69
*M. kansasii*	P21160	89.54
*M. scrofulaceum*	Q50397	87.88
*M. intracellulare* ATCC 13950	Q49575	84.55
*M. avium*	Q06947	83.33
*M. leprae TN*	P31951	81.46
*M. smegmatis* ATCC 700084/mc(2)155	A0QU51	70.64

Comparisons to *M. tuberculosis* ATCC 25177/H37Ra sequence with Clustal Omega

### Ag85B proteins of common mycobacterial pathogens exhibit distinctive tryptic peptides

Mass spectrometry can often differentiate highly homologous proteins by small differences in fragment ions observed in the MS/MS spectrum of their tryptic peptides. To examine whether such peptide differences represent a potential means to distinguish among mycobacterial species responsible for clinical infections, we analyzed the Ag85B protein sequence of *Mtb* and the three slow-growing NTM most commonly associated with pulmonary NTM infections (*M. avium*, *M. intracellulare*, and *M. kansasii*) to identify species-specific tryptic peptides (Table 2). Several of these peptides distinguished *Mtb*-derived Ag85B from homologues expressed by one or more of these NTM, with two peptide regions identifying the Ag85B homologue secreted by each of these species. Specifically, *Mtb* Ag85B peptides PGLPVEYLQVPSAAMGR, VQFQSGGNGSPAVYLLDGLR, AGCQTYK, AADMWGPSSDPAWER, NDPTQQIPK and GDLQSSLGAG were all distinguishable from the corresponding peptides of each of the three NTM species. Only the predicted *Mtb* GDLQSSLGAG and NDPTQQIPK peptides and their corresponding NTM peptide variants could identify the Ag85B protein of each species. However, predicted *M. avium* and *M. intracellulare* variants of the *Mtb* GDLQSSLGAG peptide required a cleavage event before a proline residue, which commonly occurs with greatly reduced frequency. *M. avium* and *M. intracellulare* had identical sequence for four predicted peptides (PGLPVEYLQVPSAGMGR, VQFQSGGNGSPAVYLLDGLR, AGCSTYK, and AADMWGPSSDPAWER). *Mtb* and *M. kansasii* revealed identical WETFLTSELPQWLSANR peptides, while sequence variants of this peptide distinguished Ag85B derived from *M. avium* and *M. intracellulare*. Finally, all four species had an identical SSNLK peptide.

**Table 2:** Predicted Ag85B peptide ions detectable by LC–MS/MS from mycobacteria commonly involved in pulmonary disease

Precursor ion (2+)	Peptide sequence	*M. tb*	*M. av*	*M. in*	*M. ka*
892.972	PGLPVEYLQVPSAAMGR	+			
885.964	PGLPVEYLQVPSAGMGR		+	+	
913.977	PGLPVEYLQVPSPSMGR				+
1068.048	VQFQSGGNNSPAVYLLDGLR	+			
1039.537	VQFQSGGNGSPAVYLLDGLR		+	+	
1068.540	VQFQSGGDNSPAVYLLDGLR				+
414.189	AGC[+ 57.0]QTYK	+			
393.676	AGC[+ 57.0]STYK		+	+	
400.684	AGC[+ 57.0]TTYK				+
1039.521	WETFLTSELPQWLSANR	+			+
993.496	WETFLTSELPSYLASNK		+		
1014.010	WETFLTSELPQYLASNK			+	
838.362	AADMWGPSSDPAWER	+			
837.870	ADAMWGPSSDPAWQR		+	+	
845.868	ASDMWGPSSDPAWQR				+
520.772	NDPTQQIPK	+			
394.225	LVANNTR	+			
899.963	NDPSLHIPELVGHNTR		+		
854.952	NDPSLQIPALVGNNTR			+	
895.463	NDPSLHIPELVANNTR				+
274.656	SSNLK	+	+	+	+
452.722	GDLQSSLGAG	+			
642.315	PDLQGTLGASPGGGG		+		
637.305	PDLQSALGASSGGGG			+	
494.265	GDLQASLGAR				+

*M. tb*: *M. tuberculosis; M. av*: *M. avium; M. in*: *M. intracellulare; M. ka*: *M. kansasii*

[+ 57.0] indicates the *m/z* introduced by cysteine carbamidomethylation

### LC–MS/MS analysis of Ag85B peptides differentiates common slow-growing pulmonary mycobacterial pathogens

Based on these results, it appears that LC–MS/MS analysis of Ag85B peptides from tryptic digests of mycobacterial culture supernatants is likely to distinguish between *Mtb* and NTM infections and to identify specific NTM infections. To test this hypothesis we performed a proof-of-concept study focusing on four species responsible for the majority of pulmonary infections caused by slow-growing mycobacteria: *Mtb*, *M. avium, M. intracellulare and M. kansasii*. LC–MS/MS analysis of trypsin-digested supernatants of positive mycobacterial broth cultures of these four mycobacteria detected a subset of their predicted Ag85B peptides (Table 3). LC–MS/MS analyses of the *Mtb* culture sample detected four of its nine predicted Ag85B peptides, as well as a fifth peptide LWVYCGNGTPNELGGANIPAEFLENFVR excluded by the maximum length parameter of our in silico analysis. LC–MS/MS detected the *Mtb* NDPTQQIPK peptide or a corresponding NTM peptide variant in positive liquid culture supernatants of all four species, but the *Mtb* VQFQSGGNNSPAVYLLDGLR and WETFLTSELPQWLSANR peptides and their matching NTM variants were detectable only in three of the four digests, with only the *Mtb* and *M. kansasii* samples revealing detectable levels of both peptides. Based on these results, the *Mtb* NDPTQQIPK peptide and its NTM sequence variants appear to have strong potential as discriminatory biomarkers for these four mycobacterial species. LC–MS/MS did not detect NTM variants of the *Mtb* GDLQSSLGAG peptide, however, which were the only other peptides to exhibit species-specific discriminatory power in silico. The predicted *M. avium* and *M. intracellulare* variants of this peptide require cleavage events at unfavorable sites before proline residues, but this rationale does not apply to the *M. kansasii* peptide and the reason for its absence is unclear.

**Table 3:** Ag85B peptides detected in tryptic digests of mycobacterial culture supernatants

Organism	[M + H]^+^	Charge	Peptide sequence	Intensity
*Mtb*	2135.1	2+	VQFQSGGNNSPAVYLLDGLR	6250
	2078.0	2+	WETFLTSELPQWLSANR	2280
	1040.5	2+	NDPTQQIPK	6420
	3080.5	3+	LWVYCGNGTPNELGGANIPAEFLENFVR	460
	904.4	2+	GDLQSSLGAG	3620
*M. avium*	–	–	–	–
	1798.9	2+	WETFLTSELPSYLASNK	1186
	1986.0	3+	NDPSLHIPELVGHNTR	30,428
	–	–	–	–
	–	–	–	–
*M. intracellulare*	2078.1	2+	VQFQSGGNGSPAVYLLDGLR	231
	–	–	–	–
	1708.9	2+	NDPSLQIPALVGNNTR	1260
	–	–	–	–
	–	–	–	–
*M. kansasii*	2078.0	2+	VQFQSGGDNSPAVYLLDGLR	1523
	1789.9	2+	WETFLTSELPQWLSANR	3261
	2136.1	2+	NDPSLHIPELVANNTR	3510
	–	–	–	–
	–	–	–	–

**Fig. 2:**
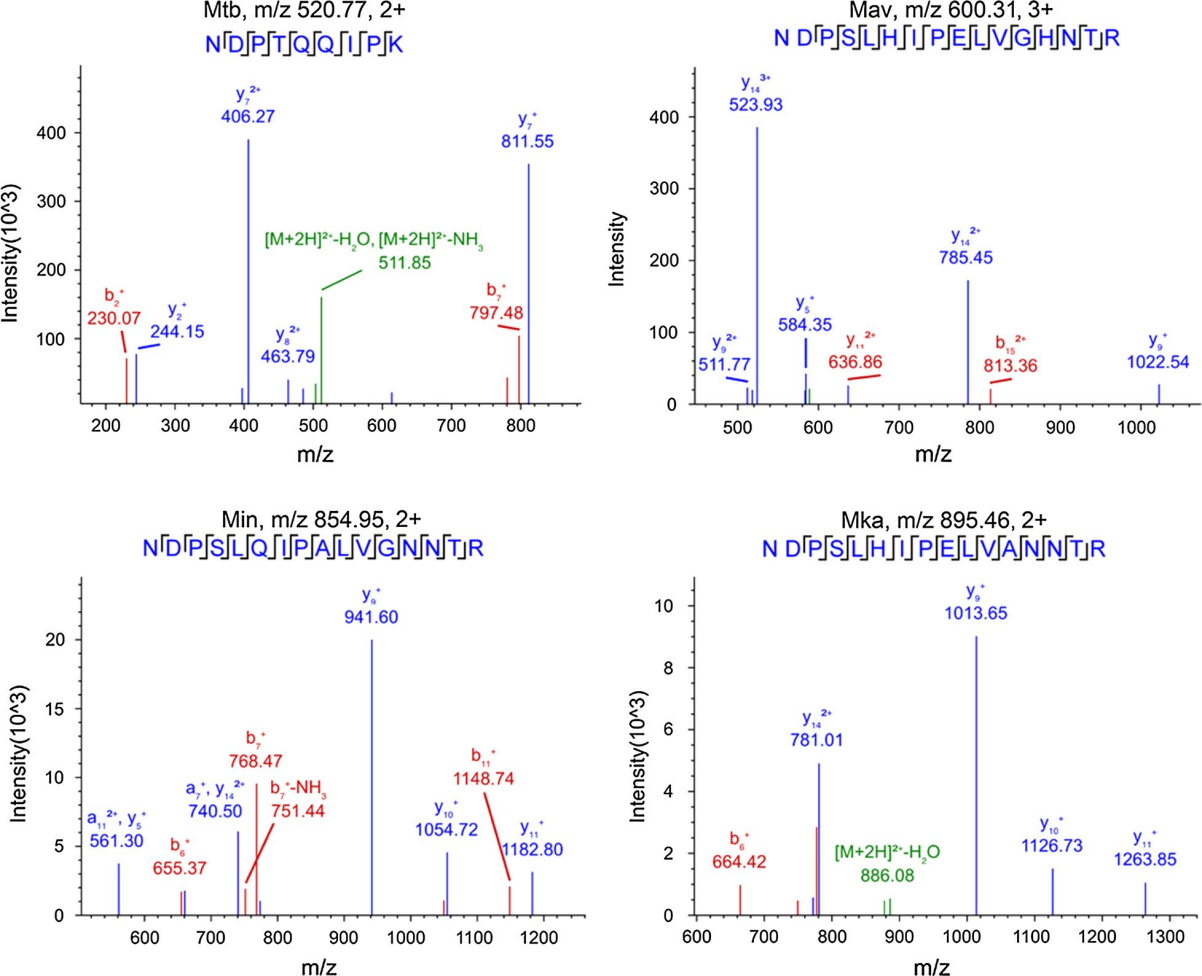
Sequence confirmation of target Ag85B peptides from mycobacterial culture supernatants by LC–MS/MS PRM mode

To confirm the identity of the predicted *Mtb* Ag85B NDPTQQIPK peptide and its corresponding NTM peptide variants, we performed an LC–MS/MS analysis using PRM mode to confirm the sequence of each of these peptides. The main transition ions for these peptides resolved at similar retention times in PRM or DDA mode LC–MS/MS analyses performed using the same preparation and chromatographic conditions. PRM product ions for the predicted *Mtb* NDPTQQIPK, *M. avium* NDPSLHIPELVGHNTR, *M. intracellulare* NDPSLQIPALVGNNTR and *M. kansasii* NDPSLHIPELVANNTR Ag85B peptides precisely matched their species-specific Ag85B sequence (Fig. 2). These results imply that tryptic peptides corresponding to this Ag85B region may represent a robust target for mycobacterial species identification.

## Discussion

NTM cases have dramatically increased in the US and other developed countries over the past few decades, but this emerging healthcare challenge has not yet given rise to rapid and accurate diagnostic methods that can identify specific NTM. Clinical suspicion of NTM disease can increase after ruling out pulmonary *Mtb* infection, so that false-negative *Mtb* results can promote mistaken NTM diagnoses and the use of inappropriate mycobacterial treatment regimens. Even accurately diagnosed NTM cases present clinical challenges, since several common NTM species exhibit different responses to available anti-mycobacterial therapy regimens. A rapid assay that accurately distinguishes between pulmonary TB and NTM cases, and identifies common NTM species responsible for pulmonary infections, would thus be of great value for rational treatment design. In the present work, we demonstrate that LC–MS/MS analysis of Ag85B peptides from early mycobacterial broth cultures provides rapid and accurate species identification.

Researchers attempting to develop *Mtb*-diagnostic immunoassays for use with clinical isolates have examined the utility of several *Mtb*-derived antigens, including Ag85 complex proteins, CFP-10/ESAT-6 [28], MPT64 [29] and the phospholipid lipoarabinomannan (LAM) [30]; however, most of these factors are not appropriate for NTM species identification. *Mtb, M. kansasii, M. marinum* and *M. szulgai* express CFP-10/ESAT-6, but other clinically important NTM, including mycobacterium avium complex members, do not [31]. MPT64 is not a reliable marker for active TB cases since *Mtb mpt64* deletions and mutations can cause false-negative assay results [32], whereas LAM is a phospholipid and cannot reliably differentiate among mycobacterial species.

Ag85B plays a pivotal role in the synthesis of mycobacterial cell walls, and while it was first identified in *Mtb,* multiple NTM express homologues, including important pathogenic NTM species such as *M. avium, M. intracellulare and M. kansasii*. Ag85B is also actively secreted and active *Mtb* cases can produce detectable levels of Ag85B in minimally invasive biological specimens, such as sputum and serum [20–23], suggesting its potential utility as a direct biomarker of mycobacterial burden. Relatively few studies have examined its diagnostic potential using patient samples, but studies using serum and cerebrospinal fluid [21, 33] have observed moderate sensitivity (82 and 79%, respectively) with moderate to good specificity (86 and 97%). Ag85 complex proteins specifically interact with fibronectin [34] and exhibit robust sequence conservation across mycobacterium species, which may affect sensitivity and specificity of their detection by immunoassay methods. One recent study isolated mycobacteria from sputum samples using an Ag85B immunomagnetic enrichment approach prior to an *Mtb*-specific PCR analysis in an attempt to improve *Mtb* detection efficiency [35]. The sensitivity of this relatively labor-intensive approach for *Mtb* cases did not surpass Xpert-MTB/RIF assays results (89.9 vs. 95.2%), and this study did not examine the diagnostic sensitivity of this method for common NTM species causing pulmonary disease. Regardless of its capacity to identify specific mycobacterium species, however, this approach cannot provide quantitative data, since it is unable to distinguish between DNA derived from viable and non-viable bacilli.

Culture remains the gold standard for laboratory diagnosis of mycobacterial infections, and while it is well documented that liquid media detects mycobacteria much earlier than solid media, rapid and accurate identification of mycobacteria from liquid broth cultures typically relies on costly commercial probes systems or time-consuming PCR and/or sequencing assays.

Our LC–MS/MS assay approach overcomes all these issues, directly detecting and identifying species-specific peptides derived from Ag85B secreted by positive mycobacterium broth cultures, to allow rapid and accurate identification of mycobacterium species. The diagnostic specificity of this approach does not suffer when used to analyze highly homologous proteins if the composition of the analyzed target peptides differs by at least one amino acid. While we have shown proof-of-principle results using supernatants from mycobacterium positive broth cultures, this method may also allow direct antigen detection in clinical samples, yielding results that could quantify bacillary burden during the response to anti-mycobacterial treatment. We have recently applied a similar approach to quantify serum levels of the actively secreted *Mtb* antigens CFP-10/ESAT-6 [36], suggesting that a variation on this method could potentially identify and quantitate species-specific Ag85B proteins in patient serum samples. The ability to diagnose and identify specific NTM infections from serum samples would represent a significant advance over current methods that require positive mycobacterial culture samples for analysis, and are thus limited by culture latency.

*Mtb*, members of the *mycobacterium avium* complex (predominantly *M. avium* and *M. intracellulare*) and *M. kansasii* are the most common slow-growing mycobacterial species found to cause pulmonary disease in most of the US [37]. In this proof-of-concept study, we identified two predicted Ag85B-derived tryptic peptides that distinguish these four species, and verified that one of these peptides accurately identified positive liquid cultures derived from each of these species. Due to the high degree of amino acid sequence conservation among mycobacterial Ag85B protein homologues, however, rare but clinically important NTM species may share target peptide sequence with these mycobacterial species. Prospective studies are required to determine the earliest time point for reliable species identification (e.g., at the first sign of broth culture positivity or before detecting growth by the continuous monitoring system), and to evaluate whether detection of Ag85B in serum samples can be used for species-specific diagnosis of NTM disease in complex clinical populations.

This approach has the potential for rapid clinical adoption since it employs methods and equipment already in use in clinical laboratories, specifically mycobacterial broth culture and mass spectrometry. While MALDI-TOF MS has shown remarkable performance for the identification of mycobacteria growing on solid media (colonies), its performance on positive mycobacterial broth (i.e. MGIT) is far less impressive. This constitutes a major limitation for the mycobacteriology laboratory, considering that mycobacterial broth cultures systems are more sensitive and detect growth earlier than solid media. Our approach mimics an ELISA method proposed to detect Mtb Ag85B in early mycobacterial culture filtrates for rapid Mtb diagnosis [23], while the high sensitivity and specificity of MS supports the idea that our Ag85B detection method can function as a fast diagnostic for NTM lung disease. Such an MS analysis approach can identify multiple mycobacterial species, and is much less subject to misdiagnosis of related mycobacteria, due to its ability to differentiate its target peptide sequence.

In summary, LC–MS/MS recognition of species-specific fragments of Ag85B from mycobacterial culture supernatants is a rapid, reliable and precise method to identify specific mycobacterial species. Future studies should address the diagnostic performance of this approach in clinical cohorts reflecting the variety of mycobacterial disease found in patient populations with a significant burden of NTM disease.

## Conclusions

Our results indicate that a subset of tryptic peptide variants produced by multiple mycobacterial species can directly identify mycobacteria present in positive liquid cultures. This study analyzed peptides derived from tryptic digests of three-week cultures of *Mtb* and three NTM responsible for the majority of pulmonary disease arising from slow-growing mycobacteria. Further analyses are necessary to determine the ability of this approach to discriminate among the spectra of mycobacteria encountered in large patient populations, the minimum time point at which mycobacterial cultures offer diagnostically useful information, and whether detection efficiency suffers in diagnostically challenging patient populations. Moreover, we hope to establish an optimized MS-based Ag85B assay that directly analyzes clinical samples, and validate its performance in a large, blinded patient cohort, to allow rapid identification of species responsible for suspected mycobacterial infections and to guide appropriate therapy regimens.

## Additional file

**Additional file 1: Table S1.** Ag85B target peptide identification and quantification by LC-MS/MS PRM mode.

## Abbreviations

AFB: acid-fast bacilli
Ag85B: antigen 85B
DDA: data dependent acquisition
IGRAs: IFNγ release assays
LAM: lipoarabinomannan
LC–MS/MS: liquid chromatography–tandem mass spectrometry
MDR-TB: multidrug-resistant TB
Mtb: *Mycobacterium tuberculosis*
NTM: non-tuberculous mycobacteria
