# Supplementary figures and images for: Antigen 85B peptidomic analysis allows species-specific mycobacterial identification
Source: Clin Proteomics. 2018 Jan 8;15:1. doi: 10.1186/s12014-017-9177-6 (PMC5757288; doi:10.1186/s12014-017-9177-6)

## Slide 1
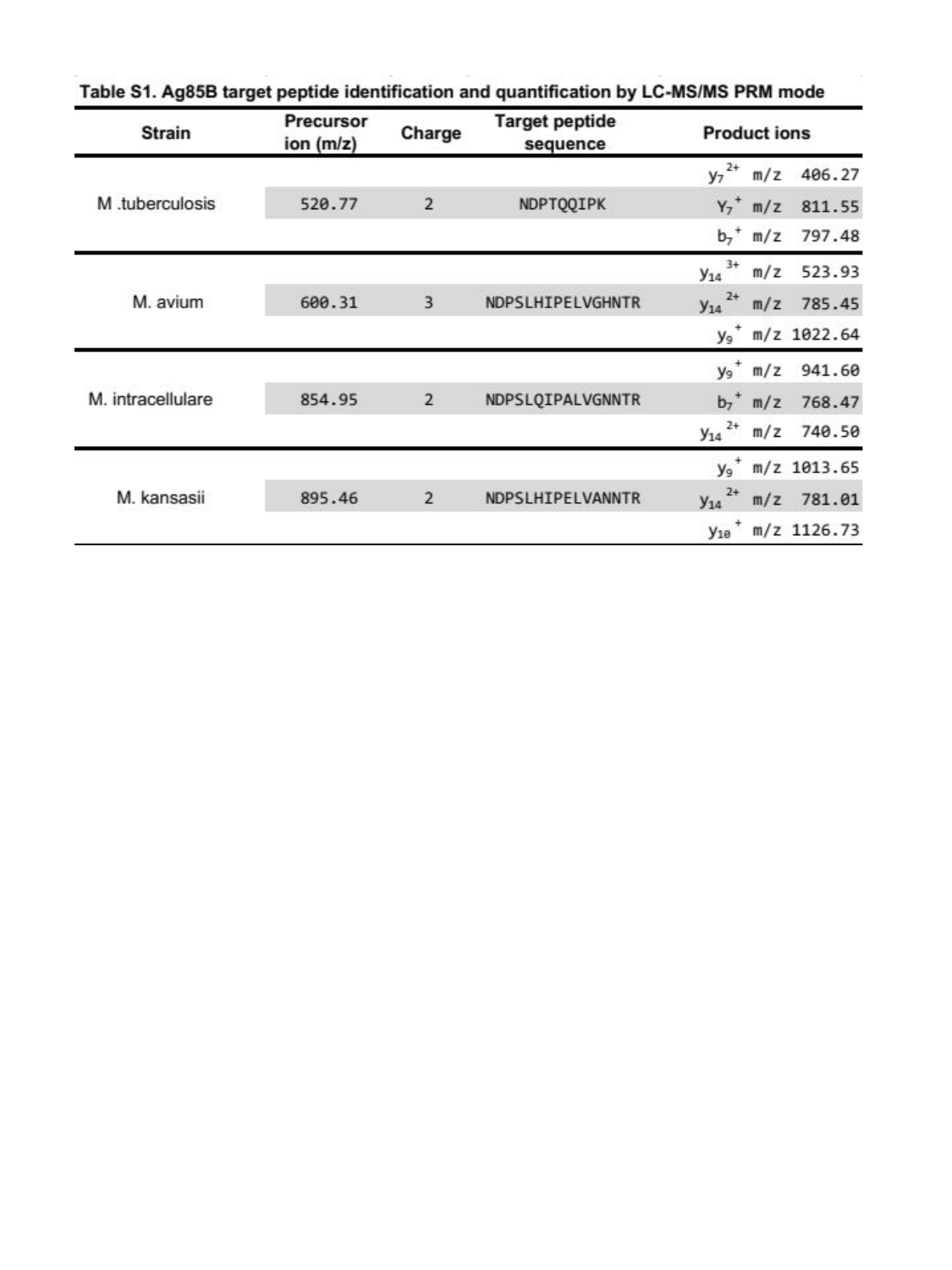

Supplement: Supplementary file 1 — Additional file 1: Table S1. Ag85B target peptide identification and quantification by LC-MS/MS PRM mode. [file 12014_2017_9177_MOESM1_ESM.pptx]
